# Plasma Pharmacokinetics of Polyphenols in a Traditional Japanese Medicine, Jumihaidokuto, Which Suppresses *Propionibacterium acnes*-Induced Dermatitis in Rats

**DOI:** 10.3390/molecules201018031

**Published:** 2015-09-30

**Authors:** Takashi Matsumoto, Yousuke Matsubara, Yasuharu Mizuhara, Kyoji Sekiguchi, Junichi Koseki, Kazuaki Tsuchiya, Hiroaki Nishimura, Junko Watanabe, Atsushi Kaneko, Kazuya Maemura, Tomohisa Hattori, Yoshio Kase

**Affiliations:** 1 Tsumura Research Laboratories, Kampo Scientific Strategies Division, Tsumura & Co., Ibaraki 300-1192, Japan; E-Mails: matsubara_yousuke@mail.tsumura.co.jp (Y.M.); mizuhara_yasuharu@mail.tsumura.co.jp (Y.M.); sekiguchi_kyouji@mail.tsumura.co.jp (K.S.); koseki_junichi@mail.tsumura.co.jp (J.K.); tsuchiya_kazuaki@mail.tsumura.co.jp (K.T.); watanabe_junko@mail.tsumura.co.jp (J.W.); kaneko_atsushi@mail.tsumura.co.jp (A.K.); maemura_kazuya@mail.tsumura.co.jp (K.M.); hattori_tomohisa@mail.tsumura.co.jp (T.H.); kase_yoshio@mail.tsumura.co.jp (Y.K.); 2 Kampo Formulations Development Center, Production Division, Tsumura & Co., Ibaraki 300-1192, Japan; E-Mail: nishimura_hiroaki@mail.tsumura.co.jp

**Keywords:** jumihaidokuto, flavonoid, tannin, genistein, liquiritigenin, Gallic acid, glucuronides, pharmacokinetics, antioxidant, dermatitis

## Abstract

Most orally administered polyphenols are metabolized, with very little absorbed as aglycones and/or unchanged forms. Metabolic and pharmacokinetic studies are therefore necessary to understand the pharmacological mechanisms of polyphenols. Jumihaidokuto (JHT), a traditional Japanese medicine, has been used for treatment of skin diseases including inflammatory acne. Because JHT contains various types of bioactive polyphenols, our aim was to clarify the metabolism and pharmacokinetics of the polyphenols in JHT and identify active metabolites contributing to its antidermatitis effects. Orally administered JHT inhibited the increase in ear thickness in rats induced by intradermal injection of *Propionibacterium acnes*. Quantification by LC-MS/MS indicated that JHT contains various types of flavonoids and is also rich in hydrolysable tannins, such as 1,2,3,4,6-penta-*O*-galloyl glucose. Pharmacokinetic and antioxidant analyses showed that some flavonoid conjugates, such as genistein 7-*O*-glucuronide and liquiritigenin 7-*O*-glucuronide, appeared in rat plasma and had an activity to inhibit hydrogen peroxide-dependent oxidation. Furthermore, 4-*O*-methylgallic acid, a metabolite of Gallic acid, appeared in rat plasma and inhibited the nitric oxide reaction. JHT has numerous polyphenols; it inhibited dermatitis probably via the antioxidant effect of its metabolites. Our study is beneficial for understanding *in vivo* actions of orally administered polyphenol drugs.

## 1. Introduction

Most orally administered polyphenols are metabolized in a living body. Flavonoids are known to undergo conjugation in the intestine and liver and circulate predominantly as glucuronides and sulfates with very little as aglycones and/or unchanged forms [1,2,3,4]. Moreover, some of polyphenols with a sugar moiety, hydrolysable tannins, and others are hydrolyzed by intestinal bacteria and then absorbed in the intestines [1,5,6,7,8]. Therefore, it is important to elucidate the metabolism and plasma pharmacokinetics of polyphenols in a study focusing on diseases of the nondigestive organs such as skin. In addition, evaluating pharmacological benefits of the metabolites identified in the systemic circulation is necessary.

Jumihaidokuto (JHT), a pharmaceutical-grade traditional Japanese (*kampo*) medicine, has been used widely for treatment of skin symptoms such as reddening, swelling, sharp pain, burning sensation, and skin diseases including acute and/or purulent skin diseases, urticaria, eczema, and athlete’s foot. JHT is reportedly effective in patients with inflammatory acne [9], and inhibited hapten-induced inflammation in a mouse model of allergic dermatitis [10]. We recently reported that JHT inhibited increases in ear thickness in two models of acute dermatitis developed using phorbol myristate acetate or *Propionibacterium acnes* (*P. acnes*), a gram-positive anaerobic microbe [11]. To investigate the potential mechanism of JHT, we examined antioxidant activities of the crude drugs and constituents, demonstrating that polyphenols originating from *Quercus cortex* (*Q. cortex*), *Schizonepetae spica* (*S. spica*), and *Glycyrrhizae radix* (*G. radix*) were active in an assay focusing on antioxidant activity against reactive oxygen species (ROS).

Consistent with our previous study [11], we hypothesized that JHT ameliorates dermatitis through antioxidant actions by the polyphenols in JHT. In this study, for the first time, we examined plasma pharmacokinetics of polyphenols present in JHT and their metabolites, and identified active metabolites contributing to its antidermatitis effect.

## 2. Results and Discussion

### 2.1. Antidermatitis Effect of JHT

**Figure 1:**
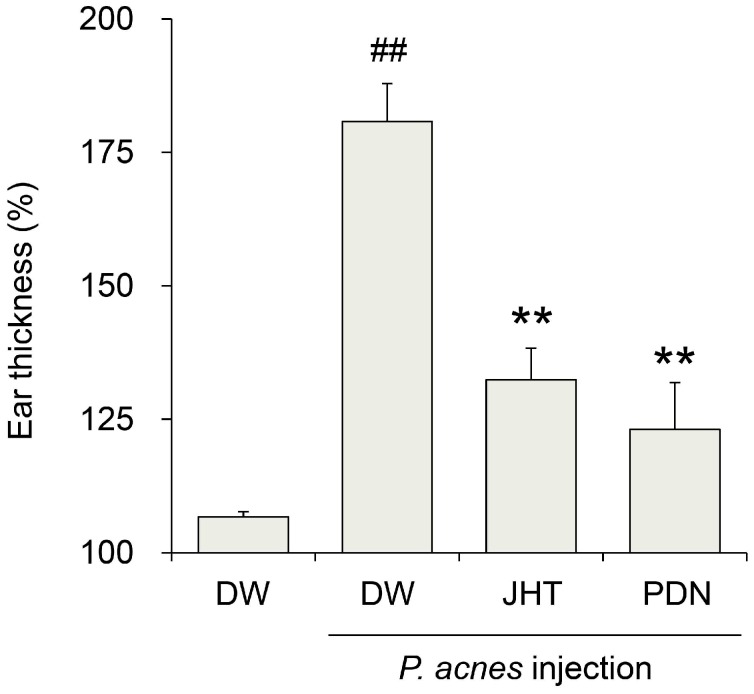
Suppression of *Propionibacterium acnes (P. acnes)*-induced dermatitis in rats by jumihaidokuto (JHT) administration. Dermatitis was induced in rats by injection of *P. acnes* (0.14 mg/50 μL/ear) into the pinna. The ear thickness was measured at 0 and 24 h after the injection. JHT suspended in distilled water (DW) was administered orally at a dose of 0.5 g/10 mL/kg at 1 h before and 6 h after the injection. Prednisolone (PDN, 10 mg/10 mL/kg) was used as a reference drug. Data represent the relative ear thickness, which was standardized against the previous values before the injection. *N* = 5. **^##^** *p* < 0.01 *vs.* saline + DW group (Student’s *t*-test), ** *p* < 0.01 significant *vs.* *P. acnes* + DW group (Dunnett’s test).

We first investigated whether JHT ameliorated *P. acnes*-induced dermatitis. Rats subjected to intradermal injection of *P. acnes* and orally administered distilled water developed rapid ear swelling with cutaneous erythema, followed by a 181% increase in ear thickness in a 24 h period compared with that prior to injection (Figure 1). On the other hand, rats treated with 0.5 g/kg JHT exhibited only a 132% increase, whereas prednisolone (PDN) exhibited a 123% increase. These results suggest that JHT has a potential antidermatitis effect.

### 2.2. Measurement of Constituents in JHT

In our previous studies, *Q. cortex*, *S. spica*, and *G. radix* exhibited strong antioxidant activities in two assays focusing on hydrogen peroxide-dependent oxidation and superoxide anion generation by xanthine oxidase [11]. The results allowed us examine these crude drugs in our dermatitis model. Only *Q. cortex* and *G. radix* were evaluated under the protocol previously reported [11], revealing that both crude drugs significantly reduced ear dermatitis by 38% and 31% inhibition, respectively, whereas JHT inhibited it by 47% inhibition. *Q. cortex* and *G. radix* appeared to be involved in the antidermatitis efficacies of JHT. We attempted to detect 23 polyphenols originating from the three crude drugs and actually identified 21 polyphenols (Table 1). The quantification analysis showed that liquiritin was the most abundant flavonoid in JHT, and the constituents such as glucoside (liquiritin apioside), aglycon (liquiritigenin), and C-ring-opened analogue (isoliquiritin) showed higher contents compared with the others. Among hydrolysable tannins, 1,2,3,4,6-penta-*O*-galloyl glucose and eugeniin were abundant. The total concentration of the 21 polyphenols was 5.1% (*w*/*w*) in JHT.

**Table 1:** Contents of polyphenols in JHT extract.

Compound Category	Subcategory	Name	Molecular Weight	Amount (mg/g JHT)
Flavonoid	Flavane	(+)-Catechin ^a^	290.08	0.077
		(−)-Epicatechin gallate ^a^	442.09	0.003
		(±)-Gallocatechin ^a^	306.07	0.011
	Flavone	Luteolin ^a,b^	286.05	0.011
	Flavonol	Quercetin ^a,b^	302.04	0.004
	Flavanone	Hesperidin ^b^	610.19	0.231
		Hesperetin ^b^	302.08	0.003
		Liquiritin ^c^	418.13	1.872
		Liquiritin apioside ^c^	550.17	1.392
		Liquiritigenin ^c^	256.07	0.298
	Chalcone	Isoliquiritin ^c^	418.13	0.130
		Isoliquiritin apioside ^c^	550.17	0.167
		Isoliquiriitgenin ^c^	256.07	0.030
	Isoflavone	Genistein ^c^	270.05	0.002
Hydrolysable tannin	Gallotannin	Hamamelitannin ^a^	484.09	0.024
		1,2,3,6-tetra-*O*-galloyl glucose ^a^	788.11	0.052
		1,2,3,4,6-penta-*O*-galloyl glucose ^a^	940.12	0.345
	Ellagitannin	Eugeniin ^a^	938.10	0.213
		1-Desgalloyleugeniin ^a^	786.09	0.030
		Castalagin ^a^	934.07	0.078
Other	Unit of tannin	Gallic acid ^a^	170.02	0.151

Methanol/purified water extracts of dried JHT (Lot No. 332031900) were analyzed by LC-MS/MS. Compounds marked by ^a–c^ originated from *Q. cortex*, *S. spica*, and *G. radix*, respectively.

This is the first report of a study to measure contents of individual constituents in JHT, showing that JHT has numerous flavonoids and is abundant in catechins with a galloyl moiety and hydrolysable tannins such as gallotannin and ellagitannin. According to the present and previous studies [1,12,13,14], the tested polyphenols are mainly from the following three crude drugs: *Q. cortex*, *S. spica*, and *G. radix.*

### 2.3. Metabolism and Pharmacokinetics of Polyphenols in JHT

**Figure 2:**
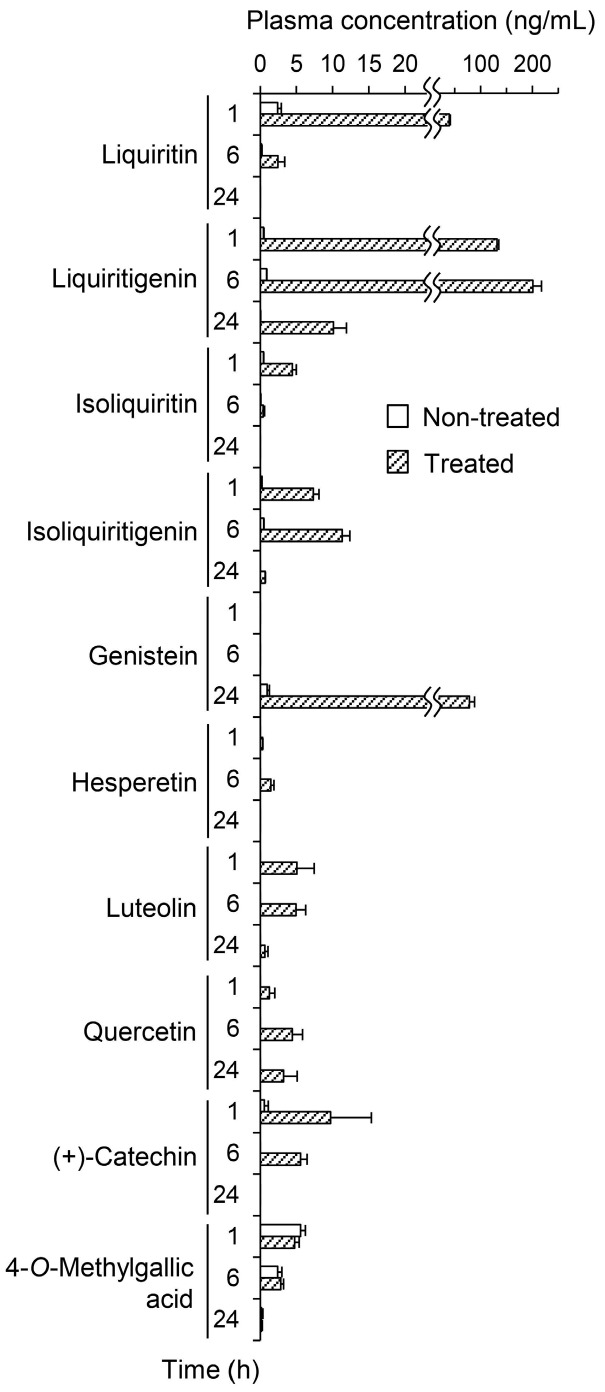
Plasma concentrations of jumihaidokuto (JHT) polyphenols in plasma before and after enzymatic hydrolysis with β-glucuronidase. Plasma samples were obtained at 1, 6, and 24 h after JHT administration to rats (2 g/10 mL/kg). The polyphenol content in the plasma were measured by LC-MS/MS before and after β-glucuronidase treatment. Each data point represents the mean ± S.E. of triplicates.

The first step in determining the plasma pharmacokinetics of the polyphenols in JHT was to quantify their intact forms in plasma from JHT-treated rats. Figure 2 shows the plasma concentrations at 1, 6, and 24 h before and after β-glucuronidase treatment. Flavonoid concentrations were very low at all time points in non-treated plasma. However, the enzymatic treatment increased concentrations of almost all flavonoids. Liquiritin, liquiritigenin, and genistein appeared as conjugated forms in the plasma and significantly increased from 2.40 to 39.1 (1 h), from 0.873 to 200 (6 h), and from 0.975 to 78.3 (24 h) ng/mL, respectively. The concentrations of hydrolysable tannins described in Table 1 were below the quantification limits. Gallic acid, a minimal unit of hydrolysable tannins, was not detected in the plasma (<10 ng/mL). However, 4-*O*-methylgallic acid, a methylated metabolite of Gallic acid, was quantified in the plasma at 5.57 ng/mL (1 h), whereas the concentration was unchanged by β-glucuronidase treatment.

**Figure 3:**
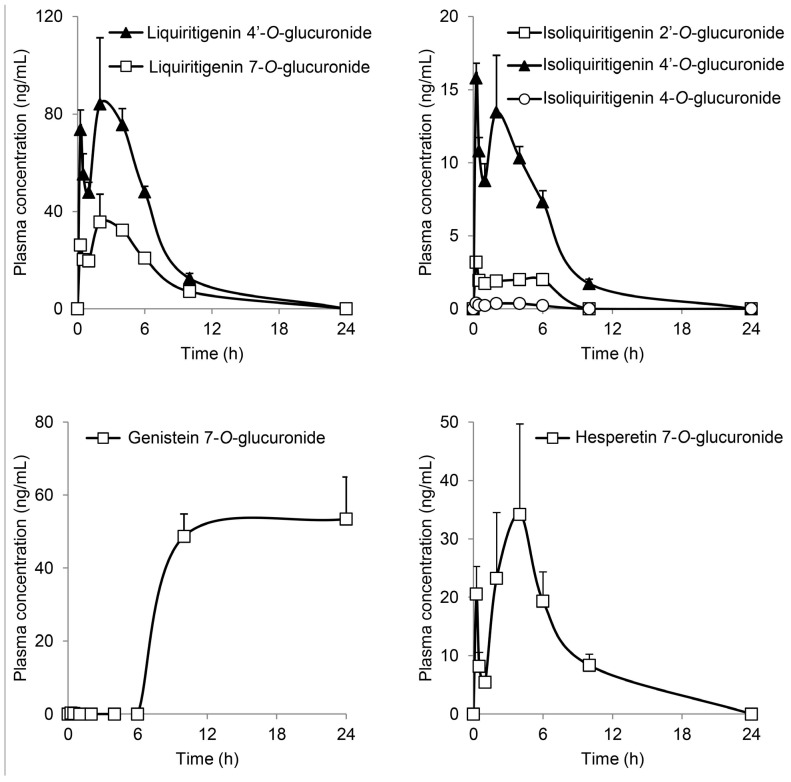
Time course of flavonoid glucuronides in the plasma of JHT-treated rats. Glucuronides of liquiritigenin, isoliquiritigenin, genistein, and hesperetin were measured in the plasma after JHT administration by LC-MS/MS. Each data point represents the mean ± S.E. of triplicates.

Focusing on flavonoid glucuronides, liquiritigenin 4′-*O*-glucuronide (LQG-4′G), liquiritigenin 7-*O*-glucuronide (LQG-7G), isoliquiritigenin 2′-*O*-glucuronide (ILQG-2′G), isoliquiritigenin 4′-*O*-glucuronide (ILQG-4′G), isoliquiritigenin 4-*O*-glucuronide (ILQG-4G), hesperetin 7-*O*-glucuronide (HPT-7G), luteolin 7-*O*-glucuronide (LTN-7G), genistein 4′-*O*-glucuronide (GEN-4′G), genistein 7-*O*-glucuronide (GEN-7G), and quercetin 3-*O*-glucuronide (QCN-3G) were examined in another set of animal studies (Figure 3). To the best of our knowledge, our study is the first to determine the overall pharmacokinetics of the glucuronides of liquititigenin and isoliquiritigenin in plasma using standard glucuronides. Various structural changes, such as opening of the C-ring during metabolism, occur in these flavonoids [15]; therefore, it was important to perform pharmacokinetic studies of these compounds. The *C*_max_ of LQG-4′G was 84.1 ng/mL, which was more than that of LQG-7G. In addition, the profiles of liquiritigenin glucuronides showed bimodality, and the first peaks appeared very rapidly (0.25 h) after JHT administration. The profiles of isoliquiritigenin and hesperetin glucuronides showed a time course similar to that of liquitritigenin glucuronides. The profiles may have resulted from the coexistence of aglycones and their glycosides in JHT; the first and second peaks may have been derived from an aglycone and glycoside, respectively.

Plasma GEN-7G significantly increased to approximately 50 ng/mL at 10 and 24 h, but did not start to increase until 6 h after JHT administration, although genistein is reported to be promptly absorbed into the bloodstream [4]. The phenotype was different from the other flavonoid glucuronides. GEN-4′G, LTN-7G, and QCN-3G did not appear in the plasma during the experiment. Our quantification study of JHT extract showed that JHT contained very little genistein and its glucoside (genistin and sophoricoside) as possible sources of GEN-7G. Thus, we speculated that JHT contains genistein derivatives modified by malonylation, acetylation, and/or another type of glycosylation [16,17,18]; intestinal bacteria needs time to generate genistein. We will perform further studies to identify the source of GEN-7G. Genistein is subjected to the enterohepatic circulation, resulting in a delayed clearance from the bloodstream [3]. Several investigators have demonstrated that the pharmacokinetics of genistein glucuronides, including GEN-7G, reveals no accumulative properties [4,19].

Table 2 shows the pharmacokinetic parameters of the detected flavonoid glucuronides and 4-*O*-methylgallic acid.

**Table 2:** Pharmacokinetic parameters of flavonoid glucuronides and 4-*O*-methylgallic acid.

Compound	*t*_1/2_ (h)	*t*_max_ (H)	*C*_max_ (ng/mL)	AUC*_0–last_* (ng·h/mL)
Genistein 7-*O*-glucuronide	-	24.0	53.4	812
Liquiritigenin 4′-*O*-glucuronide	2.27	2.00	84.1	522
Liquiritigenin 7-*O*-glucuronide	2.72	2.00	35.6	223
Isoliquiritigenin 2′-*O*-glucuronide	-	0.250	3.19	11.6
Isoliquiritigenin 4′-*O*-glucuronide	2.25	0.250	15.8	80.8
Isoliquiritigenin 4-*O*-glucuronide	5.68	0.250	0.388	1.84
Hesperetin 7-*O*-glucuronide	2.99	4.00	34.2	190
4-*O*-Methylgallic acid	1.62	2.00	6.65	32.7

-: Not calculated.

### 2.4. Antioxidant Activities of Metabolites of Polyphenols in JHT

We examined antioxidant activities of the polyphenols and their metabolites appearing in the plasma (Table 3). The IC_50_ values of GEN-7G, ILQG-4′G, ILQG-2′G, LQG-7G, and HPT-7G were 0.638, 0.691, 0.704, 0.765, and 4.27 μg/mL, respectively, whereas LQG-4′G and 4-*O*-methylgallic acid were not detectable even at the highest concentration. Only 4-*O*-methylgallic acid, but not all flavonoid glucuronides, weakened in the nitric oxide reaction, with an IC_50_ of 3.59 μg/mL. In addition, glycyrrhetic acid, a metabolite of glycyrrhizic acid originating from *G. radix*, and cimifugin from *Saposhnikoviae radix* (*S. radix*), are easily absorbed into the bloodstream [1,20]. In the rat plasma used in the present study, we actually confirmed that glycyrrhetic acid and cimifugin were identified by major peaks (Supplementary Figure S1). The two compounds were also evaluated as reference compounds of non-polyphenols. Consequently, both showed no activity.

**Table 3:** Antioxidant activity of metabolites absorbed in plasma.

	Antioxidant activity, IC_50_ (μg/mL)	
	Hydrogen Peroxide	Nitric Oxide
Genistein 7-*O*-glucuronide	0.638	n.d.
Liquiritigenin 4′-*O*-glucuronide	n.d.	n.d.
Liquiritigenin 7-*O*-glucuronide	0.765	n.d.
Isoliquiritigenin 2′-*O*-glucuronide	0.704	n.d.
Isoliquiritigenin 4′-*O*-glucuronide	0.691	n.d.
Hesperetin 7-*O*-glucuronide	4.27	n.d.
4-*O*-Methylgallic acid	n.d.	3.59
Glycyrrhetic acid	n.d.	n.d.
Cimifugin	n.d.	n.d.

All samples were evaluated at 0.1, 0.3, 1, 3, 10, or 30 μmol/L. *N* = 3. The IC_50_ values were calculated from curves constructed by plotting antioxidant activity (%) *vs.* test sample concentration. Data are shown as IC_50_ (μg/mL). n.d.: Not detectable at the maximal concentration (30 μmol/L). Namely, 4-*O*-methylgallic acid, 5.52 μg/mL; Hesperetin 7-*O*-glucuronide, 14.4 μg/mL; Genistein 7-*O*-glucuronide, 13.4 μg/mL; Liquiritigenin 7-*O*-glucuronide; Liquiritigenin 4′-*O*-glucuronide; Isoliquiritigenin 2′-*O*-glucuronide; Isoliquiritigenin 4′-*O*-glucuronide, 13.0 μg/mL; Glycyrrhetic acid, 14.1 μg/mL; and Cimifugin, 9.19 μg/mL.

**Figure 4:**
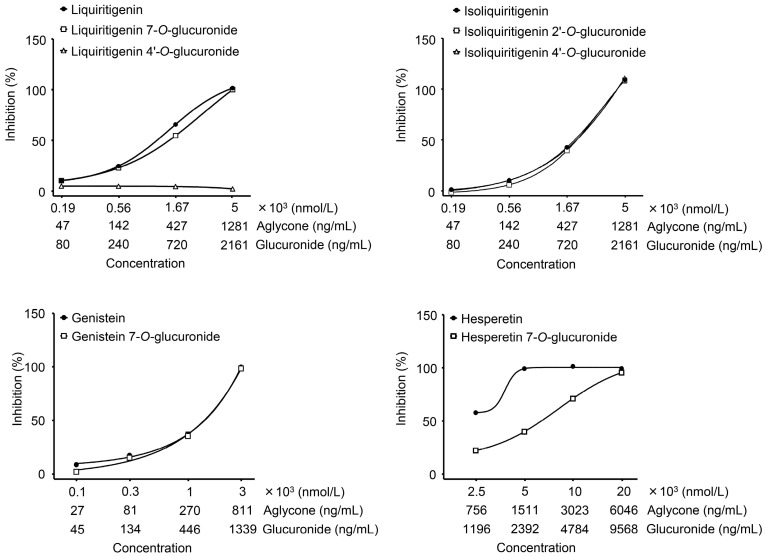
Concentration dependency of flavonoid glucuronides and comparison with aglycones. Glucuronides and aglycones of flavonoid were assayed at serially-diluted concentrations to assess on hydrogen peroxide-dependent oxidation. Test samples were incubated with hydrogen peroxide for 5 min, followed by mixing with a probe of oxidant activity. After 15 min incubation, the hydrogen peroxide-dependent fluorescent intensity was measured. Data are shown as mean ± S.E. of triplicates.

We finally tested whether there was a difference between conjugates and aglycone to understand how much the glucuronidation process affects antioxidant activities. The results showed that anti-ROS activities of LQG-7G, ILQG-4′G, and GEN-7G were completely identical to that of their aglycones (Figure 4). However, HPT-7G concentration was partially decreased and LQG-4′G showed no activity.

**Figure 5:**
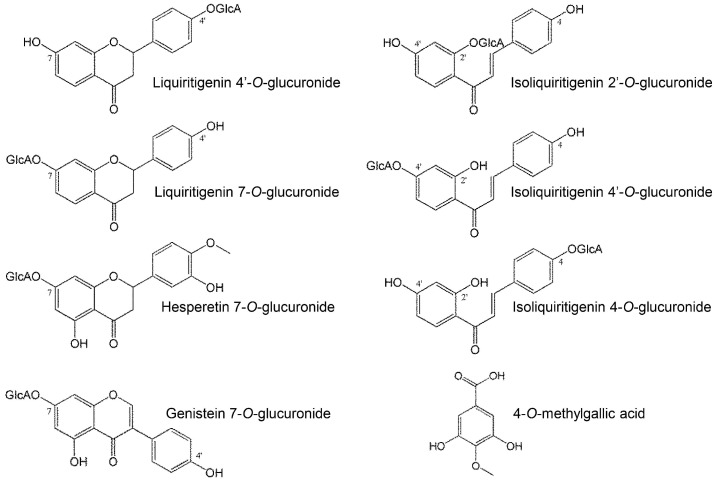
Chemical structures of metabolites appearing in plasma.

The chemical structures of the metabolites in Table 2 are shown in Figure 5. According to the studies on structure-activity correlations in flavonoids [2,21,22], the hydroxyl group of C4′-position of the B-ring is critical for expression of antioxidant and antiradical activities, and glucuronidation of the C4′-position remarkably diminishes those activities. In contrast, glucuronidation of the C7 position of the A-ring does not affect it. In our study, liquiritigenin, which showed strong activity, was deactivated by glucuronidation of the C4′ position (LQG-4′G), but not the C7 position (LQG-7G). Isoliquiritigenin and genistein were also unchanged by glucuronidation to the A-ring.

Although LQG-4′G showed the highest concentration of all of the glucuronides in the plasma, it exerted no activity. Oral administration of liquiritigenin was reported to inhibit carrageenan-induced edema in an animal model of acute inflammation [23]. Therefore, a metabolite from liquiritigenin (LQG-7G) may contribute to the antidermatitis effect of JHT. GEN-7G was thought to be the most potent on effects of JHT because it showed a high concentration in the plasma and strong anti-ROS activity. GEN-7G was absorbed at *C*_max_: 53.4 ng/mL, *t*_max_: 24 h. The IC_50_ in anti-ROS activity was 638 ng/mL. A statistically significant activity (25% inhibition) was observed at 134 ng/mL, as shown in Figure 4. There were no significant differences between the *C*_max_ and bioactive concentrations *in vitro*. It is easy to imagine that many other metabolites and intact constituents including unknowns may enter the bloodstream. It is important to consider efficacies of JHT as a whole in combination with individual effects of JHT metabolites.

As reported previously, tannins were minimally absorbed into the bloodstream [2,24]. Tannins in JHT were not detected in the plasma, although there was a problem with their detection sensitivity. Orally administered gallotannin and ellagitannin are hydrolyzed by tannases in intestinal bacteria, resulting in significant generation of Gallic acid and/or ellagic acid [7,24]. Gallic acid is metabolized mainly to 4-*O*-methylgallic acid in rats [25]. Therefore, our study used 4-*O*-methylgallic acid and demonstrated its antioxidant activity. Several studies have reported that oral administration of Gallic acid evoked various benefits including anti-inflammatory, antinociceptive, and antiedematogenic effects and that 4-*O*-methylgallic acid has various biological activities [8,26,27,28]. Gallic acid metabolites 4-*O*-methylgallic acid, 3-*O*-methylgallic acid, and 3,4-*O*-dimethylgallic acid were reported to be identified in the urine of volunteers who ingested black tea [29]. We examined the potential of 3-*O*-methylgallic acid in antioxidant assays, and it showed a strong activity against ROS but not nitric oxide (data not shown). More emphasis is needed on the pharmacological aspect of Gallic acid and its derivatives.

### 2.5. Pharmacological Characteristics of JHT Showing Antioxidant Effects

ROS are formed constitutively by mitochondria and NADPH oxidase, and their production is augmented by inflammation. It has been recently reported that ROS upregulates caspase expression and activation of the Nod-like receptor protein 3 inflammasome during inflammation [30]. Nitric oxide is produced by nitric oxide synthase, and it plays various biological roles such as neurotransmitter and vascular tone controller through activation of guanylate cyclase. In the case of an infectious response, phagocytic cells such as neutrophils produce large quantities of ROS and reactive nitrogen species (RNS) to kill foreign microbes. Excessive release of extracellular ROS/RNS damages surrounding healthy cells, and large numbers of dead neutrophils and cellular debris can cause secondary inflammation [31,32]. The pathophysiological roles of radical molecules have been well studied in models of ischemia-reperfusion injury [33,34]. This implies that ROS/RNS are critical to upregulate microvascular circulation in order to increase blood flow during an emergency following a decrease in oxygen concentration. As reported previously [35], a histological analysis of inflamed ears injected with *P. acnes* showed evidence of abscesses in which numerous neutrophils were accumulated (data not shown) 24 h after the bacterial injection. It is plausible that excessive release of ROS/RNS occurred at the inflamed ears due to *P. acnes* injection, followed by damage of vessel endothelial cells and vasodilation. Local ROS/RNS may have been significantly involved in *P. acnes*-induced ear thickness and rubefaction, although other inflammatory factors such as histamines and bioactive lipids were also involved. In the models of dermatitis caused by UV-irradiation, increased radical molecules directly trigger signs of acute inflammation such as skin edema and rubefaction [36]. Antioxidant agents, including ascorbic acid and botanical flavonoids, have been reported to prevent skin inflammation induced by UV-irradiation [37,38,39,40].

JHT contains flavonoid-rich crude drugs and tannin-rich one. Flavonoids inhibited ROS preferentially, and only 4-*O*-methylgallic acid inhibited the nitric oxide reaction. These findings are of interest when discussing the multipotency of the antioxidant effects of JHT. In addition, flavonoid glucuronides rarely function via direct modifications of intracellular events (such as kinase cascades and estrogen receptor activation) because glucuronides do not generally pass through cell membranes consisting of a lipid bilayer; they function mainly at extracellular sites. It is reasonable to assume that JHT suppresses only extracellular and excessive toxic radicals.

## 3. Experimental Section

### 3.1. JHT and Test Compounds

JHT powder (Lot No. 332031900), the base powder of JHT without excipients, was obtained from Tsumura & Co. (Tokyo, Japan), which manufactures it as an aqueous extract mixture of 10 crude components (percent composition shown in parentheses): *Platycodi radix* (14.3%), *Bupleuri radix* (14.3%), *Cnidii rhizoma* (14.3%), *Poria sclerotium* (14.3%), *Q.* cortex (14.3%), *Araliae cardatae* rhizoma (7.1%), *S. radix* (7.1%), *G. radix* (4.8%), *S. spica* (4.8%), and *Zingiberis* rhizoma (4.8%).

As candidate compounds of JHT, liquiritin apioside, isoliquiritin, isoliquiritin apioside, isoliquiritigenin, luteolin, genistin, (+)-catechin, (−)-epicatechin gallate, 1,2,3,6-tetra-*O*-galloyl glucose, 1,2,3,4,6-penta-*O*-galloyl glucose, eugeniin, 1-desgalloyl eugeniin, castalagin, LQG-4′G, LQG-7G, ILQG-2′G, ILQG-4′G, ILQG-4G, glycyrrhetic acid, and cimifugin were supplied by Tsumura & Co. with purities high enough to be evaluated in biological tests. The following compounds were purchased from chemical manufacturers: liquiritin, liquiritigenin, genistein, hesperidin, hesperetin, quercetin, (−)-gallocatechin, and Gallic acid from Wako Pure Chemical Industries (Osaka, Japan), GEN-4′G, GEN-7G, and HPT-7G from Toronto Research Chemical Industries (Toronto, ON, Canada), 4-*O*-methylgallic acid from ChromaDex (Irvine, CA, USA), sophoricoside from AK Scientific (Union City, CA, USA), LTN-7G from Chengdu Biopurify Phytochemicals Co. (Sichuan, China), QCN-3G from WuXi AppTec Co. (Shanghai, China), and hamamelitannin from Sigma-Aldrich (St. Louis, MO, USA).

### 3.2. Animals

Male Sprague Dawley rats were purchased from Japan SLC (Shizuoka, Japan) and used at the age of 7–8 weeks. The rats were allowed free access to water and standard laboratory food and housed at a temperature of 23 ± 3 °C, relative humidity of 50% ± 20%, and a 12-h light:12-h dark cycle, with lights on from 7:00 a.m. to 7:00 p.m. daily. All experimental procedures were performed according to the “Guidelines for the Care and Use of Laboratory Animals” of Tsumura & Co. Ethical approval of the experimental procedures was obtained from the Laboratory Animal Committee of Tsumura & Co.

### 3.3. *P. acnes*-Induced Acute Dermatitis and Measurement of Ear Thickness

The *P. acnes* strain ATCC6919 was purchased from Microbiologics Inc. (St. Cloud, MN, USA) and cultured in GAM broth (Nissui Pharmaceutical Co., Tokyo, Japan) in an anaerobic atmosphere using a GasPak system (Mitsubishi Gas Chemical Co., Tokyo, Japan). To prepare the *P. acnes* suspension for dermatitis experiments in rat ears, *P. acnes* was harvested, washed with phosphate-buffered saline (PBS), and centrifuged at 10,000 *g* for 10 min. After washing twice with PBS, *P. acnes* was heat-killed at 95 °C for 5 min, freeze-dried, and stored at −80 °C until use.

*P. acnes*-induced dermatitis was induced using the procedure described previously with a minor modification [35]. Heat-killed *P. acnes* (0.14 mg in 50 μL saline) or saline (control) was intradermally injected into the ventral side of both ears using a microsyringe (Hamilton Co., Reno, NV, USA) under isoflurane anesthesia. JHT was orally administered to rats at 0.5 g/kg in distilled water 1 h before and 6 h after the bacterial injection. PDN (Shionogi Pharmaceutical Co., Osaka, Japan) was orally administered to rats at a dose of 10 mg/kg in distilled water. The ear thickness was measured using a dial thickness gauge micrometer (Ozaki MFG Co., Tokyo, Japan) at 0 and 24 h after the injection. All data on the increase in ear thickness were expressed as a percentage of the previous value in each individual rat, and as the mean ± S.E. Statistical significance was evaluated by one-way analysis of variance, followed by the Dunnett’s multiple comparisons or unpaired Student’s *t*-test. A *p*-value of <0.05 was considered significant.

### 3.4. Measurement of Constituents in JHT by LC-MS/MS

Preparation of analytical samples and composition of LC-MS/MS systems were described in the previous report [12], *i.e.*, the constituents were extracted twice from 100 mg of JHT powder by 4 mL of methanol/purified water (75:25, *v*/*v*) and 4 mL of methanol/purified water (50:50, *v*/*v*) at sequential two-step. The extract solution, undiluted or diluted 10- or 100-fold with methanol, was injected into the two LC-MS/MS systems after pooling with a respective internal standard solution, niflumic acid (Sigma-Aldrich) or vincamine (Tokyo Chemical Industry Co., Tokyo, Japan). Analytical conditions are summarized in Supplementary Tables S1 and S2.

### 3.5. Pharmacokinetic Analysis of Constituents in JHT and Metabolites by LC-MS/MS

JHT prepared in water was orally administered to 16-h fasted rats weighing 230‒285 g at a dose of 2 g/10 mL/kg (*n* = 3 each). The higher dose than that in the pharmacological test was used to detect more constituents. Blood was withdrawn from the abdominal inferior vena cava with a heparinized syringe at 0.25, 0.5, 1, 2, 4, 6, 10, or 24 h after the administration. Plasma was obtained by centrifugation at 1700 *g* for 15 min at 4 °C and stored at −80 °C until use. In some experiments to deconjugate glucuronidated polyphenols, the samples were incubated with 75 mmol/L phosphate buffer containing 200 units/mL β-glucuronidase from *Escherichia coli* (Sigma-Aldrich) for 2 h at 37 °C.

For quantification of liquiritin, liquiritigenin, isoliquiritin, isoliquiritigeniin, hesperidin, hesperetin, castalagin, and all flavonoid glucuronides, 25–60 µL of standard solution and 25 µL of internal standard solution were added to 200–300 µL of plasma samples, followed by mixing well. Methanol (750–800 µL) was added to the solutions, followed by mixing and centrifugation (7000 *g*, 5 min). The supernatants were collected and dried at 40 °C under a stream of nitrogen gas. Then, the dried residue was dissolved in 50–100 μL of the HPLC mobile phase of each analytical method, and a 1–20 μL portion was injected into the LC-MS/MS systems. For quantification of genistein, luteolin, quercetin, (+)-catechin, (−)-epicatechin gallate, (±)-gallocatechin, Gallic acid, 4-*O*-methylgallic acid, hamamelitannin, 1,2,3,6-tetra-*O*-galloyl glucose, 1,2,3,4,6-penta-*O*-galloyl glucose, eugeniin, and 1-desgalloyl eugeniin, 200 μL of plasma samples were used. The amounts were measured using the same procedure, as described previously, except ethyl acetate (500 μL) and 2% acetic acid (100 μL), instead of methanol, were used concurrently as an extraction solution.

Plasma pharmacokinetic data were analyzed by non-compartmental modeling using Phoenix WinNonlin (version 6.3, Certara L.P., St. Louis, MO, USA) to determine various pharmacokinetic constants including the maximum concentration (*C*_max_), time to maximum concentration (*t*_max_), apparent elimination half-life (*t*_1/2_), and area under the plasma concentration-time curve from time zero to the last observation (AUC*_0–last_*). The *t*_1/2_ was divided by log_e_2/*k*e, where *k*e is the terminal elimination (at least three data points on the descending linear limb) rate constant.

### 3.6. Antioxidant Assays

To assess ROS, OxiSelect In vitro ROS/RNS Assay kits (R & D Biosystems, Minneapolis, MN, USA) were used according to manufacturer’s instructions. Briefly, a test sample was incubated with 2.5 μmol/L of hydrogen peroxide for 5 min, and dichlorodihydrofluorescin DiOxyQ (DCFH-DiOxyQ) was added as a probe of oxidant activity. After 15 min incubation, the hydrogen peroxide-dependent fluorescent intensity was measured using a fluorescent plate reader (Infinite M200; Tecan, Wako Pure Chemical Industries). Percentage of inhibition of ROS was calculated using the following equation:

[1 − (sample RFU^a^ − base RFU^b^)/(control RFU^c^ − base RFU^b^)] × 100 (1)

where RFU is a relative fluorescent unit of reaction solution, a is RFU of DCFH-DiOxyQ with hydrogen peroxide and sample, b is RFU of DCFH-DiOxyQ alone, and c is RFU of DCFH-DiOxyQ with hydrogen peroxide.

To quantify nitric oxide, a test sample was incubated at 37 °C for 150 min in PBS supplemented with 30 μmol/L of (±)-(*E*)-4-ethyl-2-[(*E*)-hydroxyimino]-5-nitro-3-hexenamide (NOR3). Nitric oxide generated naturally from NOR3, was measured using the Griess reagent (1% sulfanilamide, 0.1% *N*-(1-naphthyl) ethylenediamine dihydrochlride in 5% H_3_PO_4_). Briefly, 100 μL of the reacted solution was incubated for 5 min with 100 μL Griess reagent. Optical densities at 540 nm were then measured.

## 4. Conclusions

In this study, we conducted the following: (1) identification of polyphenols in JHT; (2) pharmacokinetic analysis of the polyphenols and metabolites; and (3) biological evaluation of the absorbed metabolites in assays associated with the development of dermatitis to identify active metabolites of JHT. JHT was found to contain various polyphenols that appeared to inhibit dermal inflammation via antioxidant effects of their metabolites, including GEN-7G and LQG-7G. GEN-7G is an active metabolite of JHT, based on a comparison of the absorbed concentrations and range of bioactive concentrations. Our results contribute to an understanding of the mechanisms of oral polyphenols, crude drugs, and diets containing beneficial polyphenols.

## Supplementary Materials

Supplementary materials can be accessed at: http://www.mdpi.com/1420-3049/20/10/18031/s1.
