# Supplementary material for: Plasma Pharmacokinetics of Polyphenols in a Traditional Japanese Medicine, Jumihaidokuto, Which Suppresses Propionibacterium acnes-Induced Dermatitis in Rats
Source: Molecules. 2015 Sep 30;20(10):18031–46. doi: 10.3390/molecules201018031 (PMC6332076; doi:10.3390/molecules201018031)
Supplement: Supplementary file 1 [file molecules-20-18031-s001.pdf]

Supporting Information

Table S1. LC-MS/MS methods: Ion parameters of test compounds.

Compound Name	Q1Mass (*m/z*)	Q3Mass (*m/z*)	DP (volts)	CE (volts)	CXP (volts)	#
Hamamelitannin	483.18	168.8	−105	−42	−11	1-1
1,2,3,6-Tetra-*O*-galloyl glucose	787.213	168.9	−155	−70	−13	1-1
1,2,3,4,6-Penta-*O*-galloyl glucose	939.262	769.1	−165	−44	−21	1-1
Eugeniin	937.241	301	−175	−64	−17	1-1
1-Desgalloyl eugeniin	785.268	300.7	−175	−56	−19	1-1
(+)-Catechin	288.917	109.1	−90	−32	−17	1-2
(−)-Epicatechin gallate	441.043	168.9	−80	−28	−13	1-2
(−)-Gallocatechin	305.057	125.1	−95	−30	−19	1-2
Luteolin	284.85	132.3	−90	−68	−23	1-2
Quercetin	300.98	150.7	−105	−30	−9	1-2
Genistin	431.012	269.1	−55	−16	−15	1-2
Genistein	268.919	132.7	−105	−42	−21	1-2
Gallic acid	168.79	125	−65	−22	−19	1-3
4-*O*-Methylgallic acid	182.851	123.9	−65	−30	−19	1-4
Liquiritin	417.007	254.8	−85	−28	−5	1-5
Liquiritin apioside	549.216	135	−145	−62	−23	1-5
Liquiritigenin	254.895	118.9	−80	−38	−1	1-5
Isoliquiritin	416.971	255	−100	−24	−15	1-5
Isoliquiritin apioside	549.213	254.9	−150	−40	−23	1-5
Isoliquiritigenin	254.868	119	−75	−42	−21	1-5
Sophoricoside	431.061	268	−110	−44	−15	1-6
Hesperidin	609.238	301.1	−120	−38	−19	1-6
Hesperetin	301.101	164.2	−105	−34	−9	1-6
Liquiritigenin 4′-*O*-glucuronide	431.02	113	−80	−28	−19	1-6
Liquiritigenin 7-*O*-glucuronide	431.049	113	−75	−28	−17	1-6
Isoliquiritigenin 2′-*O*-glucuronide	431.041	254.8	−80	−26	−23	1-6
Isoliquiritigenin 4′-*O*-glucuronide	431.031	254.7	−85	−28	−15	1-6
Isoliquiritigenin 4-*O*-glucuronide	431.1	135	−85	−30	−1	1-6
Genistein 4′-*O*-glucuronide	445.06	113.1	−90	−26	−17	1-6
Genistein 7-*O*-glucuronide	445.072	269.1	−85	−30	−19	1-6
Hesperetin 7-*O*-glucuronide	477.016	301.1	−85	−34	−19	1-6
Luteolin 7-*O*-glucuronide	461.029	284.8	−90	−34	−15	1-6
Quercetin 3-*O*-glucuronide	477.029	301	−75	−32	−17	1-6
Castalagin	935.025	468.9	181	39	22	2-7
Niflumic acid (IS)	280.826	236.8	−55	−24	−11	1-1, 1-2, 1-3, 1-4, 1-5
			−20	−30	−15	1-6
Vincamine (IS)	355.257	337.1	96	29	18	2-7

#: LC-MS/MS system and HPLC method ID are described in Table S2. Q1: quadrupole 1, Q3: quadrupole 3, DP: declustering potential, CE: collision energy, CXP: collision cell exit potential.

Table S2. LC-MS/MS methods: HPLC conditions.

LC-MS/MS System	HPLC Method	HPLC Condition
1	1	Column: Ascentis Express RP-amide column (100 × 2.1 mm I.D., 2.7-μm particle size; Supelco Analytical, Inc., Tokyo, Japan)<br>Mobile phase (A) 0.2 vol % acetic acid, (B) acetonitrile containing 0.2 vol % acetic acid<br>Gradient elution program (% B in A):<br>0–8 min, 22%; 8–8.01 min, 22% → 90%; 8.01–13 min, 90%; 13–13.01 min, 90% → 22%; 13.01–18 min, 22%<br>Other conditions were: flow rate, 0.2 mL/min; column temperature, 40 °C
	2	Column: Ascentis Express RP-amide column<br>Mobile phase (A) 0.2 vol % acetic acid, (B) acetonitrile containing 0.2 vol % acetic acid<br>Gradient elution program (% B in A):<br>0–5 min, 22%; 5–10 min, 22% → 90%; 10–15 min, 90%; 15–15.1 min, 90% → 22%; 15.1–25 min; 22%<br>Other conditions were: flow rate, 0.2 mL/min; column temperature, 40 °C
	3	Column: Ascentis Express RP-amide column<br>Mobile phase (A) 0.2 vol % acetic acid, (B) acetonitrile containing 0.2 vol % acetic acid<br>Gradient elution program (% B in A):<br>0–2 min, 22%; 2–10 min, 22% → 80%; 10–15 min, 80%; 15–15.1 min, 80% → 22%; 15.1–20 min; 22%<br>Other conditions were: flow rate, 0.2 mL/min; column temperature, 40 °C
	4	Column: Ascentis Express RP-amide column<br>Mobile phase (A) 0.2 vol % acetic acid, (B) acetonitrile containing 0.2 vol % acetic acid<br>Gradient elution program (% B in A):<br>0–10 min, 10%; 10–10.1 min, 10% → 80%; 10.1–16 min, 80%; 16–16.01 min, 80% → 10%; 16.01–21 min, 10%<br>Other conditions were: flow rate, 0.2 mL/min; column temperature, 40 °C
	5	Column: Inertsil Ph-3 column (100 × 2.1 mm I.D., 3-μm particle size; GL Sciences, Tokyo, Japan)<br>Mobile phase (A) 10 mM ammonium acetate, (B) acetonitrile<br>Gradient elution program (% B in A):<br>0–1 min, 20%; 1–13 min, 20% → 65%; 13–13.01 min, 65% → 20%; 13.01–18 min, 20%<br>Other conditions were: flow rate, 0.3 mL/min; column temperature, 40 °C
	6	Column: Kinetex PFP column (100 × 2.1 mm I.D., 2.6-μm particle size; Phenomenex, Torrance, CA, USA)<br>Mobile phase (A) 0.2 vol % acetic acid, (B) acetonitrile containing 0.2 vol % acetic acid<br>Gradient elution program (% B in A):<br>0–10 min, 11%; 10–30 min, 11% → 40%; 30–30.01 min, 40% → 90%; 30.01–35 min, 90%; 35–35.01 min, 90% → 11%; 35.01–40 min, 11%<br>Other conditions were: flow rate, 0.35 mL/min; column temperature, 40 °C
2	7	Column: Ascentis Express RP-amide column<br>Mobile phase (A) 0.2 vol % formic acid, (B) acetonitrile<br>Gradient elution program (% B in A):<br>0–1 min, 10%; 1–8 min, 10% → 30%; 8–10 min, 30% → 90%, 10–12 min, 90%; 12–12.01 min, 90% → 10%; 12.01–17 min, 10%<br>Other conditions were: flow rate, 0.3 mL/min; column temperature, 40 °C

LC-MS/MS system: system 1, an API4000 triple quadrupole mass spectrometer (AB SCIEX, Tokyo, Japan) equipped with an Agilent 1100 system (Agilent Technologies, Tokyo, Japan); system 2, a TripleQuad6500 (AB SCIEX) equipped with an Agilent 1290 system (Agilent Technologies).
